# Jejunal Lipoma With Intermittent Intussusception Revealed by Partial Obstructive Syndrome

**DOI:** 10.4103/1319-3767.43278

**Published:** 2008-10

**Authors:** Saâd Rifki Jai, Fatimazahra Bensardi, Farid Chehab, Driss Khaiz, Abdelmajid Bouzidi

**Affiliations:** Department of Surgery III, Ibn Rochd University Hospital, Casablanca, Morocco

**Keywords:** Intussusception, lipoma, surgery

## Abstract

Jejunojejunal intussusceptions are not very common in adults, and unlike in children, a lead point is usually found. The clinical presentations in adults tend to be more chronic or intermittent, and they include obstructive syndrome, abdominal cramps, gastrointestinal bleeding, or palpable abdominal mass at physical examination. These unspecific symptoms often lead to late diagnosis after many investigations or even only after an inappropriately extensive surgery. We report the rare case of a 37-year-old female with intermittent bowel obstruction due to jejunojejunal intussusception secondary to the lipoma. The main clinical signs of this uncommon pathology are presented together with the necessary paraclinical investigations that enable surgical treatment.

Jejunal lipoma is a rare cause of adult intussusception, is usually diagnosed during surgery with the presence of mechanical obstructive syndrome.[1] Some patients may present with subacute or chronic form, leading to late diagnosis after many investigations or even after a surgery is performed.

## CASE HISTORY

**Figure 1:**
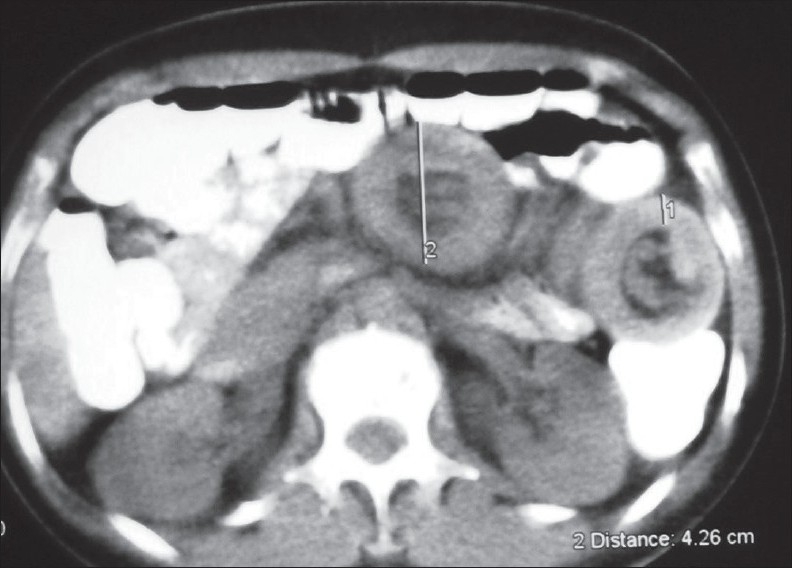
A contrast-enhanced abdominal CT scan with oral contrast administration demonstrated classic bowel-in-bowel appearance of small intestinal intussusception

**Figure 2:**
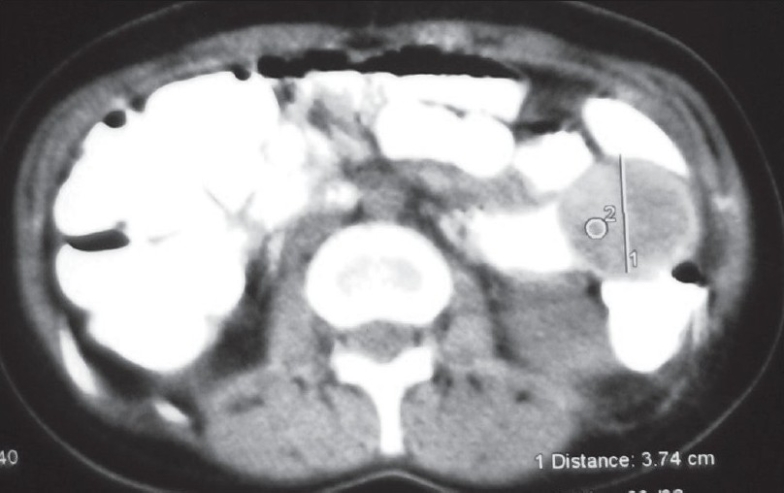
Another section of the contrast-enhanced abdominal CT scan with oral contrast administration revealed an ovoid and well-defined, low-density tumor in the intussusception

A 37-year-old female with past history of appendicectomy in 1982 and surgery for kidney lithiasis in 1996, suffering from intermittent periumbilical cramping pain with partial obstructive syndrome for nearly 3 years. During the previous 6 months (June 2006), she was operated for left ovary cyst by Pfannenstiel incision. She was admitted to the hospital on November 2006 because of increasing of abdominal pain with partial bowel obstruction. Clinical and physical examinations revealed normal vital findings with sensitive and palpable mass over the left periumbilical region. Rectal examination was normal and hematologic values were normal. There was a dilatation of a proximal small bowel loop on the plain abdominal radiograph. Ultrasonography (US) revealed multilayered small bowel lumen along the periumbilical area. A contrast-enhanced abdominal computed tomography (CT) scan with oral contrast administration demonstrated classic bowel-in-bowel appearance of small intestinal intussusception [Figure1] and an ovoid and well-demarcated low-density tumor within the lumen of the intussuscipiens [Figure 2]. The patient underwent surgical intervention; the leading point of intussusception was a 3-cm polypoid mass in the third portion of jejunum with vital intestine so that only a segmental resection was performed. Pathological examination confirmed a submucosal lipoma of the jejunum.

## DISCUSSION

Intussusception is defined as the telescoping of a proximal segment of intestine, termed intussusceptum, into the distal intestinal loop, known as intussuscipiens. The majority of intussusceptions occur in infancy and early childhood, rarely in adults (10% of all intussusceptions), and may be ileocolic, colocolic, enteroenteric, or jejunogastric without anatomic predilection.[2] The underlying causes of intussusception in adult vary greatly, a mechanical cause is found in 90% of adults. The lead points of adult intussusception that involve the colon are usually malignant (carcinoma, lymphoma), whereas those that involve the small bowel tend to be benign (lipoma, polyp, Meckel's diverticulum, sprue or from lymphoid hyperplasia secondary to viral infection).[2] Lipoma is not a common tumor in the gastrointestinal tract, and gastrointestinal lipoma may be submucosal or subserosal. Most of them are asymptomatic, although they may cause abdominal pain, bowel obstruction, and gastrointestinal bleeding. Intestinal intussusception caused by lipoma is uncommon. It is particularly rare when lipoma is located in the small intestine.[3] However, adult intussusception is a rare disease and symptoms tend to be more chronic or intermittent with vague abdominal pain (71%), nausea and vomiting (68%), abdominal distension with partial obstruction (45%), or palpable abdominal mass at physical examination;[1,2] therefore, it is difficult to diagnose the intussusception. In our case, the correct diagnosis was not achieved instantly due to this changeable clinical appearance of intussusception; however, the patient's history of colicky abdominal pain and partial obstructive syndrome was almost certainly secondary to the episodes of intermittent intussusception.

Ultrasonography is very appropriate and useful in the diagnosis of intussusception. First, it is a more available and generalized technique than CT, enabling it to be used more often with emergency and acute symptoms, and therefore, it is available at times of abdominal crisis in intermittent processes. It showed the typical multilayered appearance consisting of the alternating hyperechoic and hypoechoic concentric rings that represent alternating layers of mucosa, bowel wall, and mesenteric fat in cross section. Lipoma of the small intestine may appear on US as a highly echoic mass.[4,5] CT scan with oral and intravenous contrast has been shown to be the most accurate diagnostic tool for the evaluation of intermittent intussusception. Intussusception appears on CT scan as one of the following: an apparent mass lesion caused by a thickened segment of bowel; the intussusceptum telescoping into the intussuscipiens; a crescent-like, eccentric low-attenuation fatty mass, representing entrapped mesenteric fat; a rim of contrast material encircling the intussusceptum, representing coating of the opposing bowel walls of the intussusceptum and intussuscipiens; air bubbles peripheral to the upper part of the intussusception similar to the contrast material, which may enter between the opposing bowel walls; and a leading mass. Lipoma of the small intestine may appear on CT scans as a round, homogeneous, well-circumscribed mass with fat density. Ultrasonography has a similar sensitivity and specificity to that of CT.[2,4,5] Abdominal conventional radiographs may show dilated bowel, a paucity of bowel gas in the right lower quadrant, or a soft-tissue mass that produces a concave defect in the air of the intestine.[2]

Surgery is usually the treatment of choice for symptomatic gastrointestinal lipomas with laparotomy and resection of the affected intestinal segment. Laparoscopic surgery is an excellent alternative approach, and can be a useful method for reducing hospital stay, complications, and return to normal activity.[1,6]

## CONCLUSION

Jejunojejunal intussusception caused by lipoma is a rare disease. Ultrasonography is a safe, noninvasive and effective tool for early, preoperative diagnosis of patients with intussusception and acute abdominal pain. Computer tomography is sensitive to delineate the nature of lipoma as well as the reluctant intussusception.
